# Effects of Prenatal Consumption of Caprine Milk Oligosaccharides on Mice Mono-associated with *Bifidobacterium Bifidum* (AGR2166)

**DOI:** 10.2174/1874285801711010105

**Published:** 2017-06-30

**Authors:** Caroline Thum, Kikuji Itoh, Wayne Young, Adrian Cookson, Warren McNabb, Nicole Roy

**Affiliations:** 1 Food Nutrition & Health Team, Food & Bio-based Products Group, AgResearch Grasslands, Palmerston North 4442, New Zealand; 2 Riddet Institute, Massey University, Palmerston North 4442, New Zealand; 3 Laboratory of Veterinary Public Health, Graduate School of Agricultural and Life Sciences, University of Tokyo, Tokyo 113-0033, Japan; 4 Food Assurance & Meat Quality Team, Food and Bio-based Products Group, Hopkirk Institute, Palmerston North, New Zealand

**Keywords:** Maternal diet, Caprine milk oligosaccharides, Bifidobacteria, Germ-free mice, Mono-associated, Prebiotic

## Abstract

**Background:**

Prenatal consumption of oligosaccharides are associated with changes in the maternal gastrointestinal tract (GIT) microbiota with health consequences for the offspring. It has previously been demonstrated that caprine milk oligosaccharides (CMO) stimulate the growth and fermentation rate of *Bifidobacterium bifidum* AGR2166.

**Objective:**

The objective of this study was to examine the effects of *B. bifidum* AGR2166 and prenatal consumption of CMO, alone or in combination, on the dam’s large intestine, foetal development and ability of *B. bifidum* to translocate from the gastrointestinal lumen to organs and foetal membranes.

**Method:**

Germ-free BALB/c mice, inoculated with *B. bifidum* AGR2166 or anaerobic phosphate buffer, were fed either diet supplemented with CMO or with galacto-oligosaccharide. Pregnant mice were euthanised 1 to 3 days before the expected delivery date and samples collected for analysis.

**Results:**

Dietary CMO, regardless of bifidobacterial inoculation was shown to increase GIT weight and to reduce foetal weight compared to galacto-oligosaccharide-fed dams. *B. bifidum* AGR2166 DNA was detected in the mesenteric lymph nodes, liver, plasma and placenta of the dam by amplification of the bifidobacterial 16S rRNA gene.

**Conclusion:**

*B. bifidum* AGR2166 DNA was detected in maternal organs, however there is no indication that live bifidobacteria was able to translocate during pregnancy. Further studies using conventionally-raised mouse models will develop a deeper understanding of the interactions between dietary CMOF, the host, and bacteria.

## INTRODUCTION

Prenatal consumption of a specific prebiotic and/or probiotic is associated with changes in the maternal gastrointestinal tract (GIT) microbiota [1] and metabolism [2], with implications for both maternal and neonatal health [3]. These effects are likely to involve selective fermentation of commensal bacteria, release of fermentative products, improvement of barrier function of the GIT epithelium and mucosal immune regulation. Evidence that orally administered probiotic strains to the mothers can translocate from the GIT to the umbilical cord blood [4] and meconium [5] also suggest direct effect of maternal GIT on the foetus development.

Caprine milk oligosaccharides (CMO) are potential prebiotic oligosaccharides, with some degree of similarity to those found in human milk oligosaccharides (HMO) [6]. HMO are natural oligosaccharides known to stimulate the establishment of the neonatal GIT microbiota by accelerating the development and the maturation of the neonate’s GIT (morphologically and immunologically [7];). We have previously demonstrated that CMO enriched fraction (CMOF) was able to stimulate *in vitro* the growth and fermentation rate of bifidobacteria strains isolated from the faeces of exclusively breast-fed infants [8]. Of the isolated strains, *Bifidobacterium bifidum* (AGR2166) were shown to efficiently ferment CMOF [8] producing acetic and lactic acid. Another study, from our group showed that consumption of CMO by the dams during gestation and lactation improved the development of the pups, and increased the relative abundance of bifidobacteria and butyric acid in the colon, at weaning [9].

Therefore, the objective of this study was to examine the effects dietary CMOF alone or in combination with inoculation of *B. bifidum* (AGR2166) on maternal GIT morphology, caecal fermentation and foetal development. The ability of *B. bifidum* to translocate from the GIT lumen to the maternal organs and foetal membranes was also investigated.

## METHODS

### Animals and Diet

This study was approved by the Tokyo University Animal Ethics committee, Tokyo, Japan. Germ-free (GF) BALB/c mice (32 female and 16 male) (Laboratory of Veterinary Public Health, The University of Tokyo), between 15 and 23 weeks of age, were housed in a vinyl isolator sterilised with 2% peracetic acid under normal conditions (12 h light/dark cycle). Two different diets, AIN-76A supplemented with CMOF containing 0.9% CMO (treatment; CMO diet) and AIN-76A supplemented with galacto-oligosaccharide (GOS), at the same concentration that are present in the CMOF (GOS diet), were formulated to meet mouse nutritional requirements (Oriental Yeast Co. Ltd., Japan) (Table **1**). Maltodextrin concentration was adjusted in the CMO and GOS-modified diets for nutritional balance. Dietary oligosaccharide composition was analysed by liquid chromatography–mass spectrometry and dietary GOS was analysed by high performance ion chromatography as previously described [6].

Mice were randomly assigned to 4 groups (GF/GOS diet, GF/CMO diet, Bifidobacteria/GOS diet, Bifidobacteria/CMO diet) of twelve mice each (eight females and four males). Mice were inoculated with *B. bifidum* AGR2166 (0.5 mL of bacterial suspension containing approximately 10^8^ CFU/mL), or 0.5 mL of anaerobic phosphate buffer solution via oral gavage and fed either GOS or CMO diet. Microbiological analysis of fresh faecal samples collected on days 3, 10 and 17 of the experimental period and caecal digesta after euthanasia was used to evaluate *B. bifidum* AGR2166 colonisation.

The mice were euthanised 1 to 3 days before the expected date that pups would be delivered and samples were collected for analysis. Maternal organs were weighed and blood, liver, large intestine mesenteric lymph nodes (MLN), uterus, amniotic fluid, foetus and foetal blood were aseptically collected. Approximately half of each sample was stored at -80°C for DNA extraction, and half plated on a BL agar plate (a selective medium for *Bifidobacterium* spp [10]). Plates were incubated anaerobically at 37°C and *B. bifidum* growth assessed after 48 h. The GIT was removed, and the length-to-weight ratio recorded prior to full dissection. Proximal sections of the colon were collected for histological assessment of the colonic crypt length and goblet cell numbers.

### Colon Histology

Formalin fixed transverse, paraffin embedded sections of the colon were stained with haematoxylin and eosin. Morphology measurements were performed using bright field microscopy at 200 times magnification and Image-Pro Plus 4.0 (MediaCybernetics, Bethesda, MD, USA). Crypt lengths were determined by measuring an average of 80 random fully longitudinally sectioned crypts and number of goblet cells was determined by counting goblet cells in an average of 30 crypts per mouse.

### Caecal Short Chain Fatty Acids

The concentrations of acetic acid, propionic acid, ethyl-butyric acid, butyric acid, isovaleric acid, valeric acid, formic acid, lactic acid and succinic acid in the caecal digesta were measured using a Shimadzu RID 10A HPLC (Shimadzu Oceania Ltd., Auckland, New Zealand) fitted with a Bio-Rad Aminex 87H 9µm, 7.5 x 300 mm HPLC column, as previously described [8].

### Isolation of Bifidobacteria DNA

Total DNA was extracted from maternal plasma, MLN, spleen, liver, placenta, amniotic fluid, foetus and caecum content using DNeasy Blood & Tissue Kit (Qiagen, Biolab NZ) according to the manufacturer’s instructions for the extraction of Gram-positive bacterial DNA. Samples were boiled for 1 min or transferred to a tube containing 0.3 g of sterile 0.1 mm diameter zirconium beads (Sigma-Aldrich) and shaken in a mini bead beater (Biospec Products, Bartlesville, OK, USA) set at maximum for 45 s, followed by centrifugation at 13,000 X g for 1 min before being added to the DNeasy Mini spin column. From the isolated genomic DNA, the bifidobacterial 16S rRNA gene was amplified with bif 164 and bif 662 primers [11] by PCR using a previously described method [8]. The presence of bacterial contamination in maternal plasma and liver was determined by PCR amplification of the 16S rRNA gene using the universal bacterial primers fD1 and rD1 [12].

### Statistical Analysis

Unbalanced ANOVA was used to compare treatments (GenStat V15). The different rate of bifidobacteria translocation between treatments was evaluated by a binomial test. Statistically different means were determined using the *post hoc* LSD test at 5%.

## RESULTS

### Bifidobacteria Detection

Bifidobacterial numbers recovered from faeces remained stable (10^8^ CFU per gram of faeces) during the experimental period. Bifidobacterial numbers, in dam faeces, were not increased by the consumption of CMO during the experimental period. The presence of *B. bifidum* in the caecum of inoculated mice was also confirmed by amplification of the *B. bifidum* 16s rRNA gene. No amplification of this gene was observed in digesta from GF mice. No contamination, with other bacterial strains, were detected by PCR amplification of the 16S rRNA gene using the universal bacterial primers fD1 and rD1in both GF and *B. bifidum* inoculated mice.

### Dams and Litters

Of the eight female mice originally mated in each treatment group, few were pregnant by the end of mating period; GF/GOS, 1; GF/CMO, 3; Bifidobacteria/GOS, 4; Bifidobacteria/CMO, 3. Due to the low mating success in the experimental group GF/GOS, no comparisons were drawn between these mice and the other treatment groups.

### Body and Organs Weights and Histology

There was no evidence that diet, bifidobacteria or the interaction between diet and bifidobacteria inoculation affected maternal colon morphology and maternal body, uterus, spleen, liver and MLN weight. Dams fed the CMO diet, however, had increased GIT wet weight and lower foetus wet weight compared to dams fed the GOS diet, regardless of inoculation status (Table **2**). Dams mono-associated with bifidobacteria had lower GIT weight and conceived a higher number of foetuses compared to GF mice, regardless of the diet (Table **3**). Since there was only one observation for GF mice fed the GOS diet, no variation for this treatment could be estimated and the effects of diet and bifidobacteria were influenced by this single observation.

### Caecal Short Chain Fatty Acids

Formic acid was only detected in inoculated dams fed GOS diet (Bifidobacteria/GOS, 177 ± 10.0, mean (µmol/g) ± SE). Succinic acid concentration was increased (P=0.03) in the caecum of GF dams fed CMO compared to inoculated dams (GF/GOS, nd; GF/CMO, 163.4 ± 24; Bifidobacteria/GOS, 3.8 ± 0.8; Bifidobacteria/CMO, 21.9 ± 5, mean (µmol/g) ± SE). Lactic acid was detected in higher concentrations (P=0.02) in the caecum of the dams inoculated with bifidobacteria and fed GOS diet compared to those fed the CMO diet (GF/GOS, nd; GF/CMO, 1.3 ± 0.8; Bifidobacteria/GOS, 274.7 ± 28; Bifidobacteria/CMO, 138.7± 8, mean (µmol/g) ± SE).

### Bifidobacterial Translocation

No viable bacteria, as determined by culture, were detected in the maternal organs after 48 h incubation in anaerobic conditions. However, *B. bifidum* AGR2166 DNA was detected in samples from GOS-fed dams (1 liver, 2 plasma, 3 placenta) and CMO-fed dams (2 MLN, 2 liver, 3 plasma and 3 placenta) by amplification of the bifidobacterial 16S rRNA gene. In animals that received the CMO diet, bifidobacteria DNA was detected in a greater number of samples when compared to GOS fed animals (P=0.01). Only non-specific bands were detected in GF dam’s samples.

## DISCUSSION

To our knowledge, this is the first study to report the effects of an oligosaccharide-enriched diet fed to mono-associated dams. The low levels of fecundity observed in all treatments restricted the number of samples analysed in this study. Reduced fecundity and implantation rate have been reported as limitations for the use of GF mice as a model [13]. GF mice inoculated with *B. bifidum* AGR2166 had increased number of foetuses, in agreement with a previous study where enhanced implantation rate was observed in GF mice following bacterial colonisation [14]. CMO-fed dams had reduced foetal weight compared to GOS-fed dams, however, most GOS-fed dams analysed were inoculated with bifidobacteria. Bifidobacteria colonisation improves nutrient absorption, therefore increased nutrients may reach the foetus thereby improving growth.

CMO diet did not increase dam’s faecal bifidobacterial numbers during experimental period. This was also reported in a previous study were consumption of CMO by the dams during gestation and lactation did not increased the relative abundance of bifidobacteria in the colon but increased in the colon of the offspring at weaning (9).

The consumption of CMO, regardless of bifidobacterial inoculation, was shown to increase GIT weight. Dietary fibre [15, 16] increases water retention and mucin secretion in the small intestine and colon as a consequence of both short chain fatty acid (SCFA) production [17] and mechanical stimulation by increased faecal mass [18]. Bacterial colonisation may have opposite effects to that, by decreasing caecal enlargement due to the metabolism of resistant carbohydrates, as observed in this study by the oral administration of *B. bifidum* AGR2166.

*B. bifidum* AGR2166 preferentially produced lactic acid and formic acid; reaching higher concentrations when mice where fed GOS diet. Specific rate of sugar consumption plays an important role in the ratio of the final metabolites produced by *Bifidobacterium* spp. If bifidobacteria consume the energy source fast, larger amounts of lactic acid and relatively smaller amounts of acetic acid, formic acid, and ethanol are produced [19]. A higher concentration of succinic acid, was detected in the GF mice fed CMO compared to inoculated mice. Trace amounts of SCFA have previously been reported in GF mice [20], and it is likely that these arise from cellular metabolism in the mice as a result of the oligosaccharides in the CMO diet.

*B. bifidum* AGR2166 DNA was detected in the maternal plasma, liver, MLN and placenta of inoculated mice. Bifidobacteria administered orally to GF mice have been previously reported to translocate to MLN, spleen, liver, lungs and kidneys [21, 22], supporting these findings. Bacterial DNA was detected in a higher number of samples from CMO fed dams compared to those fed the GOS diet. Although this is in agreement with previous work where dietary fibre was shown to increase bacterial translocation [23], no qPCR analysis was done to confirm that. The non-detection of bacterial DNA in the amniotic fluid and foetus or viable bacteria, in all dams’ organs, may be due to the low levels of bifidobacteria in these samples. Human and laboratory mammal foetuses swallow amniotic fluid, prior to the presence of milk in the GIT, therefore, bacteria in the amniotic fluid if any, are likely to be found in the foetal GIT. Rather than an inoculum for GIT colonisation, the low levels of bacteria previously reported in umbilical cord blood, amniotic fluid, placenta and foetal membranes are likely to stimulate the development of GIT mucosal immune system of the foetus [24], in preparation for life outside the uterus.

In conclusion, consumption of CMO diet, regardless of bifidobacterial inoculation, did not modify maternal caecal fermentation and colon morphology. The reduction on foetal weight attributed to the CMO diet needs to be evaluated with caution due to the low number of animals. Further studies using conventionally-raised mouse models will develop a deeper understanding of the interactions between dietary CMOF, the host, and bacteria.

## Figures and Tables

**Table 1:** GOS and caprine milk oligosaccharide diet composition based on AIN-76A diet.

**Diet composition (g/Kg)**	**GOS**	**CMO**
**Casein**	200	200
**Corn oil**	50	50
**Cellulose, BW200**	50	50
**DL-Methionine**	3	3
**Mineral Mix S10001**	35	35
**Vitamin Mix V10001**	10	10
**Choline Bitartrate**	2	2
**Corn Starch**	500	500
**Maltodextrin**	128.1	118.1
**Test components (g/kg)**	**GOS**	**CMO**
**GOS**	1.8	2.0
**CMO**	0	9

**Table 2:** Effect of diet on dam absolute and normalised body weight, organ weight colon crypt length and goblet cells number and gastrointestinal tract length (mean ± SE, expressed in mg/g or cm/g total body weight).

Treatments*	**GOS**	**CMO**	**P-value**	**LSD**
Body weight (g)	40.0 ± 1.1	42.7 ± 11	0.3	4.2
Uterus weight (mg/g)	183 ± 17	170 ± 12	0.2	52
Number of foetus	5.7 ± 0.4	6.2 ± 0.5	0.4	1.4
Foetus weight (mg/g)	131 ± 20	98 ± 8	0.03	44
Spleen weight (mg/g)	2 ± 0.07	2 ± 0.09	0.7	0.3
Liver weight (mg/g)	4.8 ± 0.3	4.4 ± 0.2	0.2	1.0
MLN weight (mg/g)	0.4 ± 0.04	0.6 ± 0.1	0.1	0.3
GIT length (cm/g)	1.31 ± 1.3	1.35 ± 1.4	0.1	0.1
GIT weight (mg/g)	124 ± 10	136 ± 4	0.03	18
Goblet cells number	22.6 ± 1.8	19.6 ± 1.0	0.1	4.4
Crypt size (µm)	184.2 ± 7.5	175.4 ± 3.5	0.2	17.7

* GOS and CMOF containing diet were identified as GOS and CMO, respectively (n= GOS, 5; CMO, 6)

**Table 3:** Effect of bifidobacteria on body weight, organ weight, colon crypt length and goblet cells number and gastrointestinal tract length (mean ± SE, expressed in mg/g or cm/g total body weight).

Treatments*	**GF**	**Bifidobacteria**	**P-value**	**LSD**
Body weight (g)	39.8 ± 1.5	42.4 ± 1.0	0.1	4.4
Uterus weight (mg/g)	155 ± 14	188 ±13	0.2	54
Number of foetus	5.0 ± 0.4	6.6 ± 0.3	0.003	1.5
Foetus weight (mg/g)	90 ± 6	126 ± 16	0.1	47
Spleen weight (mg/g)	2.08 ± 0.04	2.02 ± 0.09	0.6	0.3
Liver weight (mg/g)	4.3 ± 0.3	4.8 ± 0.2	0.4	1.1
MLN weight (mg/g)	0.6 ± 0.1	0.5 ± 0.05	0.6	0.3
GIT length (cm/g)	1.39 ± 0.04	1.30 ± 0.03	0.1	0.1
GIT weight (mg/g)	147 ± 3	121 ± 6	0.02	19
Goblet cells number	18.5 ± 1.1	22.3 ±1.2	0.07	4.3
Crypt size (µm)	170.8 ± 2.6	184.3 ± 5.2	0.1	16.7

* Treatments are identified as GF and Bifidobacteria for non-inoculated and inoculated mice respectively (n= Bifidobacteria, 7; GF, 4)
